# Association of Medicare Advantage Premiums With Measures of Quality and Patient Experience

**DOI:** 10.1001/jamahealthforum.2022.2826

**Published:** 2022-08-26

**Authors:** Amelia M. Haviland, Sai Ma, David J. Klein, Nathan Orr, Marc N. Elliott

**Affiliations:** 1 Carnegie Mellon University, Pittsburgh, Pennsylvania; 2 RAND Corporation, Santa Monica, California; 3 Humana Inc, Louisville, Kentucky

## Abstract

**Importance:**

Quality of care varies substantially across Medicare Advantage plans. The price information that Medicare Advantage enrollees are most likely to consider when selecting a Medicare Advantage plan is the monthly premium. Enrollees may select plans to minimize premium or, alternatively, use premium as a proxy for quality and select plans with higher premiums; however, quality implications of these choices are unknown.

**Objective:**

To determine the extent to which the quality of care offered by Medicare Advantage plans varies within vs across premium levels.

**Design, Setting, and Participants:**

This was a retrospective cross-sectional study of the population enrolled in Medicare Advantage plans in 2016 to 2017 using clinical quality measures from the Healthcare Effectiveness Data and Information Set (HEDIS), patient experience measures from the Consumer Assessment of Healthcare Providers and Systems (CAHPS) surveys, and administrative data. Data were analyzed from March 2021 to March 2022.

**Exposures:**

Medicare Advantage monthly premium.

**Main Outcomes and Measures:**

Ten publicly reported 2017 HEDIS measures and 5 publicly reported 2017 CAHPS measures linearly transformed to a 0 to 100 scale.

**Results:**

The 168 968 Medicare Advantage CAHPS respondents were representative of the enrollee population (14% were <65 years old and eligible through disability; 24% ≥80 years old; sex and race/ethnicity data were not considered); 40% were in 591 plans with no monthly premiums and less than 6% were in 144 plans with monthly premiums of $120 or more. There were from 77 054 to 2 139 422 enrollees by HEDIS measure. Among all Medicare Advantage enrollees, 79% were in plans with either a $0 premium or a low monthly premium (≤$60); patient experience and clinical quality were generally similar in these 2 categories of plans. To a small extent, enrollees in moderately high ($60-$120) and high (≥$120) premium plans reported better patient experience (+1.4 [95% CI, 0.7-2.1] and 2.2 [95% CI, 1.5-2.9] points) and received better clinical care (1.4 [95% CI, 0.3-2.5] to 3.3 [5% CI, 2.1-4.5] percentage points on most measures than those with $0 and low-premium plans. Quality differences within each premium level category were substantial; the within-premium category plan-level SDs were 6.5 points and 7.2 percentage points for patient experience and clinical quality, respectively. A plan at the 50th percentile of clinical quality and patient experience in the high premium category would fall in the 65th and 62nd percentile within the $0-premium category, respectively.

**Conclusions and Relevance:**

This population-based cross-sectional study found that although quality of care and patient experience were slightly higher with higher-premium plans, quality varied widely within each premium category. High-quality care and patient experience were found in each price category. Thus, paying higher premiums is not necessary for higher quality care in Medicare Advantage plans. Greater engagement of enrollees and advocates with quality of care and patient experience information for Medicare Advantage plan selection is recommended.

## Key Points

**Question:**

To what extent does the quality of care offered by Medicare Advantage plans differ across vs within monthly premium levels?

**Findings:**

This retrospective cross-sectional study found statistically significant but small-to-medium sized (1-3 points of 100) improvements for most clinical and patient experience quality measures with higher premiums. There was a negative association for 1 measure; in contrast, at each premium level, there was substantial variation (≥5 points) in the quality of care among Medicare Advantage plans.

**Meaning:**

These findings suggest that although there were modest improvements in the mean quality of care offered by high-premium Medicare Advantage plans, plans with high quality of care are available at every premium level.

## Teaser

This population-based cross-sectional study measures the extent to which Medicare Advantage enrollees experience higher quality of care and have better patient experiences in higher-premium plans.

## Introduction

Health care costs are a concern for many people, from patients to policy makers. Patients facing monthly insurance premiums and payments at the point-of-care often struggle to fit health care costs into their budgets.^1^ To our knowledge, the question of whether there are quality risks for lower-price options and quality benefits for paying more is unresolved. Consumers’ perceptions of price and quality are particularly important when choosing health insurance. Reliable and comparable quality indicators are often lacking,^2^ and some consumers are skeptical of plans’ quality ratings given the complexity of the calculations.^3^ Conversely, insurance plan prices are often standardized to facilitate price comparison, unlike prices in medical settings. Therefore, price, rather than quality, may dominate plan selection decisions.

Medicare Advantage, which encompasses more than one-third of people with Medicare,^4^ is a potentially informative setting for comparing plan pricing and quality. Unlike fee-for-service Medicare coverage, Medicare Advantage prices and quality are monitored and publicly reported by the Centers for Medicare & Medicaid Services (CMS).^5^ Plan prices have several components, including deductibles, subsidies, and a maximum out-of-pocket expense. Most Medicare Advantage enrollees who participated in focus groups reported that they mainly consider the prices of premiums and out-of-pocket expenses and do not consider information on the quality of plans.^6^ These focus group findings are supported by empirical research that found that premiums explained enrollment twice as strongly as quality ratings.^7^ Hence, Medicare Advantage plans provide a unique opportunity to examine the associations between prices salient to plan choice and quality.

A number of studies examine patient perspectives on price and quality. A 2016 nationally-representative survey of patient perceptions found that although most respondents did not think there was a relationship between quality and prices, a substantial minority (approximately 25%) did believe there was a positive association.^8^ Research on observed, rather than perceived, associations between quality and price for medical care have had inconsistent findings. A systematic review found that approximately one-third of studies reported positive associations (higher cost associated with higher quality), one-third reported negative associations, and another one-third found no association.^9^ Another recent systematic review reported no general association between hospital price and quality of care, but this seemed to depend on the condition being treated and the specific resources being used. When process instead of outcome measures are used, more studies tend to detect significant positive associations between price and quality.^10^

In this study, we took the perspective of a Medicare Advantage enrollee who focused on the monthly premium when choosing a Medicare Advantage plan, and we consider the plan quality that might be expected at each premium level; taking both the mean and variation in plan quality into account. The quality measures we investigated are publicly reported and are comprised in the CMS Quality Bonus Payment pay-for-performance system: 10 Healthcare Effectiveness Data and Information Set (HEDIS) measures of clinical quality and 5 measures of enrollees’ experiences with health care based on the Consumer Assessment of Healthcare Providers and Systems (CAHPS) surveys. We hypothesized that any association between Medicare Advantage plan premium and quality would be weak for both clinical quality and patient experience measures, with plans of highly variable quality available at each premium level.

## Methods

This study was approved by the RAND Corporation (Santa Monica, CA) Institutional Review Board. We followed the Strengthening the Reporting of Observational Studies in Epidemiology (STROBE) reporting guidelines (eAppendix D in the Supplement provides how each guideline was addressed by this study).

### Data Collection

We used data from the following 3 sources: (1) the 2017 Medicare Advantage CAHPS survey, reflecting enrollees’ 2016 health care experiences; (2) 2017 HEDIS data, reflecting 2016 clinical quality of care; and (3) 2016 CMS administrative data on plan features, including premium and other plan- and insurer-level characteristics. While CAHPS and HEDIS measures both represent the Medicare Advantage population, they have different eligibility criteria and are distinct data sets. The Medicare Advantage CAHPS survey is a nationally representative survey of Medicare Advantage enrollees’ health care experiences and receipt of influenza (flu) immunizations during the prior 6 months. We used data from 168 750 enrollees of Medicare Advantage plans that also offered prescription drug coverage (Table 1) and for which information on plan-level premiums (benefit packages) was available (further details are available in eAppendix C in the Supplement). Analytic weights that adjust for probability of selection into the sample residing in the 50 US states or the District of Columbia, propensity to respond, and post-stratification to match each Medicare Advantage contract’s enrolled population were used in all analyses of CAHPS measures.^11,12^ The Medicare Advantage CAHPS informational materials and telephone scripts indicate that participation in the survey is voluntary and that informed consent to the survey is implicit in returning a completed survey.

**Table 1.** Descriptive Characteristics of Plans and Enrollees by Premium Level, 2017 MA CAHPS Survey

Characteristic	Monthly premium, $				
	0	>0 to 60	>60 to <120	≥120	All
Plans, No.	591	693	279	144	1707
Enrollees, % (weighted)	43.2	35.3	15.7	5.7	100
Respondents, No.	58 685	75 122	21 612	13 331	168 750
**Enrollees, weighted %**					
Age, y					
18-64	11	20	10	7	14
65-74	48	42	41	33	44
75-79	18	16	21	21	18
≥80	23	21	27	39	24
Education					
No high school degree	19	25	13	11	20
High school graduate/some college	59	58	62	63	60
4-y College degree or more	22	16	25	25	21
Health					
Excellent/very good	36	31	36	35	34
Good	37	36	38	41	37
Fair/poor	27	34	26	24	29
Mental health					
Excellent/very good	56	49	58	60	54
Good	28	30	29	28	29
Fair/poor	16	21	13	12	17
Any proxy assistance	12	15	10	10	13
Answer proxy assistance	4	5	4	5	4
Dual status	11	41	7	4	20
Low-income supplement/not dual	5	4	4	2	4
**Benefit package**					
Plan type					
HMO	83	71	46	40	71
HMO-POS	5	6	7	19	6
Local PPO	9	16	26	37	16
PFFS	0	1	5	1	1
Regional PPO	3	6	17	3	6
Chronic SNP	3	2	0	0	2
Dual-eligibility SNP	4	34	0	0	14
Out-of-pocket maximum, $					
<3500	26	6	19	48	19
3500-6000	39	26	42	21	34
>6000	28	31	34	30	30
Data missing	7	37	5	1	17
Contract/sponsor					
Nonprofit	22	27	44	76	30

Abbreviations: CAHPS, Consumer Assessment of Healthcare Providers and Systems; HMO, Health Maintenance Organization; HMO-POS, Health Maintenance Organization with a point-of-service option; MA, Medicare Advantage; PPO, Preferred Provider Organization; PFFS, Private Fee-for-Service; SNP, Special Needs Plan.

The HEDIS measures^13^ are developed, tested, and validated under the direction of the National Committee for Quality Assurance and are gathered through surveys, medical charts, and insurance claims.^14^ A total of 2 671 165 enrollees were eligible for 1 or more of the 9 HEDIS measures available from administrative data—flu immunization (the 10th) is collected via the Medicare Advantage CAHPS Survey.

The 2016 administrative data of plan characteristics included information at 3 nested levels: plan benefit package, which we refer to as a *plan*; CMS contract (1 or more plans offered by a sponsor in a geographic area); and sponsor (ie, insurance company). Because premiums vary at the plan level, we assessed the association between plan-level quality and premiums ($0, >$0 to ≤$60, >$60 to <$120, and ≥$120 per month). Sensitivity analysis regarding these categories is available in eAppendix C, eFigure 1, and eFigure 2 in the Supplement.

### Measures

The CAHPS patient experience measures were either single-item ratings of care or composites of several related items. We focused on the composites, which have been found to be less sensitive^15,16^ to differences in use of survey rating scales by respondent characteristics. From the CAHPS survey, we obtained 5 enrollee-level patient experience measures (getting needed care, getting care quickly, care coordination, getting needed drugs, and customer service), 1 HEDIS measure (receipt of flu immunization), and enrollee-level case-mix adjustors. We also created an overall composite of patient experience measures by calculating the equally-weighted mean of the 5 patient experience measures for each respondent. All measures were linearly transformed to a scale from 0 to 100 points and differences of 1, 3, and 5 or more points for CAHPS measures on this scale were considered to be small, medium, and large, respectively.^17,18^

From the HEDIS data, we obtained information on whether each enrollee was eligible for each measure, and if so, whether they received recommended care. We focused on HEDIS measures used in the 2017 Star ratings:

- Adult body mass index (BMI; calculated as weight in kilograms divided by height in meters squared) assessment
- Breast cancer screening
- Colorectal cancer screening
- Controlling high blood pressure
- Diabetes care: HbA_1c_ control (<9%)
- Diabetes care: nephropathy
- Diabetes care: retinal eye examination
- Drug therapy for rheumatoid arthritis
- Osteoporosis management in women who had a fracture
- Influenza immunization (from the CAHPS survey)

From CMS administrative data, we obtained and summed the Part C (medical care) and Part D (prescription drug) monthly premiums for each Medicare Advantage plan. We also obtained the maximum out-of-pocket expenses and plan-level characteristics, such as plan structure or special needs plan type and contractual features, such as nonprofit status from the CMS administrative data.

The described measures comprise the Medicare Advantage Star Ratings, which aggregate these and additional measure sets and are summarized in ordinal categories at the contract level.^19^ Modeling the underlying continuous patient experience and HEDIS measures rather than the ordinal Star Ratings allowed us to determine if the premium-quality associations differed across measures, to more precisely determine the magnitude of each association, and to estimate the associations at the benefit package level.

### Statistical Analysis

#### Patient Experience Measures (CAHPS)

One primary and 5 secondary multivariate linear regression models were run with standard errors adjusted for plan-level clustering. The unit of analysis was the CAHPS survey respondent. The predictors of interest were the monthly plan premium levels. All models included indicators for geography, specified as hospital referral regions (HRRs), to address differences in prices and survey scale use by geography. All CAHPS models included standard CAHPS respondent-level case-mix adjusters to address differences in enrollee populations across plans: age, educational attainment, physical and mental health, receipt of proxy assistance,^20^ receipt of Medicaid, and receipt of a low-income subsidy; a secondary model included only these terms as a baseline (Model 0). Because health care experiences and member preferences may differ by plan structure, the main model included indicators for plan structure (eg, Health Maintenance Organization [HMO] or Preferred Provider Organization [PPO]; Table 1). Although flu immunization is a HEDIS measure, it is collected via the CAHPS survey; it shares a data structure with the CAHPS measures but is dichotomous and, like other HEDIS measures, is not case-mix adjusted in standard scoring. Results for this measure are shown separately (not included in the overall CAHPS composite; Table 2). In addition to the overall composite CAHPS measure model, the main model specification was also run separately for each of the CAHPS measures that compose the overall composite.

**Table 2.** Association Between Monthly Premium and Quality of Care (0-100 scale)^a^

Outcome	No.	Monthly premium, $				
		>0 to 60 vs 0	>60 to <120 vs 0	≥120 vs 0	>60 to 120 vs >0 to 60	≥120 vs >60 to 120
CAHPS						
Composite^b^	166 988	0.30 (0.24)	1.44 (0.34)^c^	2.18 (0.34)^c^	1.14 (0.31)^c^	0.75 (0.37)^d^
HEDIS^b^						
Composite	3 192 511	0.32 (0.47)	1.43 (0.58)^d^	3.29 (0.60)^c^	1.11 (0.55)^d^	1.86 (0.58)^e^
Osteoporosis	77 054	−3.60 (1.54)^d^	−6.41 (1.74)^c^	−6.14 (1.81)^c^	−2.81 (1.45)	0.27 (1.67)
Influenza immunization	161 411	1.94 (0.77)^d^	6.14 (0.95)^c^	10.32 (1.00)^c^	4.20 (0.91)^c^	4.19 (1.03)^c^

Abbreviations: CAHPS, Consumer Assessment of Healthcare Providers and Systems; HEDIS, Healthcare Effectiveness Data and Information Set; MA, Medicare Advantage.

^a^ All reported results are from linear regression models with standard errors (SEs) adjusted for clustering on plan.

^b^ *P* < .001 for 3df block test of premium categories for MA CAHPS composite, HEDIS composite, and influenza immunization measure model; *P* = .001 for HEDIS osteoporosis model.

^c^ *P* < .001.

^d^ *P* < .05.

^e^ *P* < .01.

We considered 4 additional models in sensitivity analyses. Model 2 tested whether the association between premium and patient experience persists when the plan maximum out-of-pocket expense was included. Model 3 included an interaction between plan premiums and for-profit status. Model 4 replaced HRR with county. Model 5 removed enrollees also enrolled in Medicaid.

#### Clinical Care Measures (HEDIS)

We obtained a summary of the relationship between premium and the HEDIS measures, which we will refer to as a composite, using a model that is described in eAppendix A in the Supplement. We ran 1 primary and 5 secondary linear regression models^21^ on the HEDIS composite with standard errors adjusted for clustering on the plan. As with the CAHPS analysis, all models included indicators for geography, specified as HRRs. The primary and secondary HEDIS models were parallel to the CAHPS models described previously, except that the HEDIS measures were not case-mix adjusted so the models did not include patient-level covariates. The same sensitivity analyses described were implemented on the HEDIS measures.

**Figure 1.**
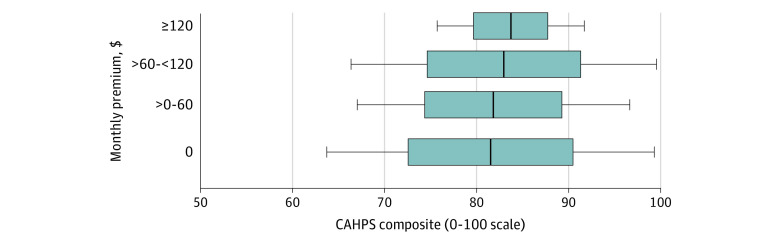
Differences in Patient Experiences Within and Between Plan Premium Categories (CAHPS composite, 0-100 scale) The gray box indicates 1 SD (67%) of plans, the whiskers indicate 2 SD (95%) of plans, and the dark line shows the mean quality. Mean quality at each premium level from Table 2 (see also eTables 2A and 2B in the Supplement); plan SDs from models in eAppendix A in the Supplement; and CAHPS indicates Consumer Assessment of Healthcare Providers and Systems.

**Figure 2.**
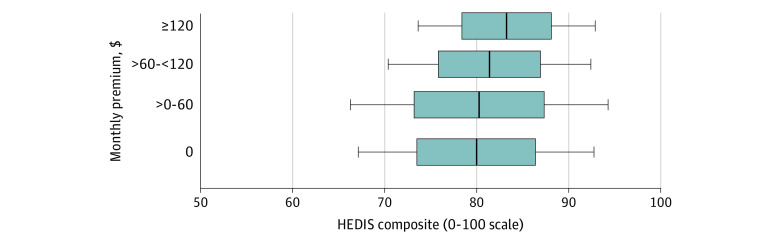
Differences in Clinical Quality Within and Between Plan Premium Categories (HEDIS composite, 0-100 scale) The gray box indicates 1 SD (67%) of plans, the whiskers indicate 2 SD (95%) of plans, and the dark line shows the mean quality. Mean quality at each premium level from Table 2 (see also eTables 2A and 2B in the Supplement); plan SDs from models in eAppendix A in the Supplement; and HEDIS indicates Healthcare Effectiveness Data and Information Set.

Supplementary analyses (Figures 1 and 2; eAppendix A in the Supplement) calculated the plan-level standard deviation of quality within premium level for the 2 aggregate measures (Medicare Advantage CAHPS and HEDIS). These within-premium level SDs were compared with the differences in adjusted mean quality across premium levels.

We did not attempt to identify a causal effect of plan premium on quality. Instead, we considered whether a plan chosen for its high or low premium was likely to have higher or lower quality.

We performed the statistical analyses from March 2021 to March 2022 using SAS, version 9.4 (SAS Institute Inc). Two-sided *t* tests of linear regression coefficients were conducted; *P* < .05 are reported.

## Results

The 168 750 Medicare Advantage CAHPS respondents were representative of the enrollee population (14% were <65 years old and eligible through disability; 24% ≥80 years old; sex and race/ethnicity data were not considered); 40% were in 591 plans with no monthly premiums and less than 6% were in 144 plans with monthly premiums of $120 or more (Table 1). Plans with different premium levels had enrollees with different characteristics. For example, both enrollees who were under 65 years old and eligible for Medicare through disability as well as those also enrolled in Medicaid were disproportionately in lower-premium plans (particularly plans with >$0-$60 premiums). Plans with the lowest premiums were mostly HMO; all Special Needs Plans were in the 2 lower-premium categories. Enrollees in plans in the 2 higher-premium categories were more likely to be 80 years or older and have higher levels of education. The plans with the highest monthly premiums (≥$120) were less often HMOs and much more often run by not-for-profit firms.

Table 2 and Figure 1 show the mean difference in the overall patient experience composite by premium level, within plan type and HRR (full model results are available in eAppendix B and eTable 2A in the Supplement). Those enrollees in plans in the 2 higher-premium categories reported better patient experiences than those in the lowest-premium category. Adjusted mean patient experiences were similar in the 2 lower-premium categories and increased monotonically across the 3 higher- premium categories. Compared with $0 premium plans, plans with $60 to $120 monthly premiums had 1.4 (95% CI, 0.7-2.1) points higher patient experience scores and plans with more than $120 monthly premiums had 2.2 (95% CI, 1.5-2.9) points higher patient experience scores, small-to-medium effect sizes.^17^ Figure 1 illustrates how these mean differences across premium categories compared with the differences in patient experiences between plans in the same premium category (eTable 2B in the Supplement shows the adjusted means by premium level derived from the Table 2 model results); most variability in plan quality was within the premium category. Plan quality variation was larger in the 3 lower-premium categories (plan SDs, 9.0, 7.5, and 8.0 points, respectively) than in the highest premium category (4.0 points); there were no very low-quality plans in the highest premium category (eTable 1 in the Supplement); however, there were high quality plans in all premium levels.

For the individual CAHPS measures, there was evidence of an increasing trend in patient experience by premium category for 4 of the 5 measures. The exception was customer service, where no association was detected (eTable 3 in the Supplement).

Table 2 and Figure 2 show the mean difference in HEDIS measure pass rates, excluding the osteoporosis measure and flu immunization, by plan premium level, within plan type and HRR (full results in eTable 2A in the Supplement). Those enrollees in plans in the 2 higher premium categories were more likely to receive recommended care than those in the lowest premium category. Rates of received recommended care were similar in the 2 lower premium categories and increased across the 2 higher premium categories. Compared with $0 premium plans, plans with $60 to $120 monthly premiums had 1.4 (95% CI, 0.3-2.5) percentage points (pp) higher rates of receiving recommended care and plans with monthly premiums of $120 or more had 3.3 (95% CI, 2.1-4.5) pp higher rates of receiving recommended care. Figure 2 illustrates how these mean differences across premium categories compared with the differences in rates of receiving recommend care between plans in the same premium category; again, most variability in plan quality was within premium category (plan SDs, 5.0-7.0 pp) and plans with high (>90%) or low (<75%) rates of providing recommended care were found in each premium category (eTable 1 in the Supplement).

Effects were larger for flu immunization and rates increased for each premium level. Compared with $0 premium plans, flu immunization was 1.9 (95% CI, 0.4-3.4), 6.1 (95% CI, 4.2-8.0), and 10.3 (95% CI, 8.3-12.3) pp higher in plans with monthly premiums of $60 or less, more than $60 to less than $120, and $120 or more, respectively.

The osteoporosis management measure showed a distinct relationship with plan premium; rates of receiving this recommended care were *lower* in higher premium plans. Compared with $0 premium plans, osteoporosis management rates were 3.6 (95% CI, 0.6-6.6), 6.4 (95% CI, 3.4-9.4), and 6.1 (95% CI, 2.6-9.6) pp lower in plans with monthly premiums $60 or less, more than $60 to less than $120, and $120 or more, respectively.

eTable 4 in the Supplement shows evidence of higher levels of clinical care in higher premium Medicare Advantage plans for 4 of the 8 HEDIS measures included in the HEDIS composite model. These 4 measures were colorectal cancer screening, breast cancer screening, diabetes eye exam, and drug therapy for arthritis, where either the top 2 or all 3 premium categories that were more than $0 had higher quality than the $0 premium category.

eTables 5 to 8 in the Supplement show the results of all model specifications and sensitivity analyses for the overall composite patient experience measure, the pooled HEDIS measure, and the 2 single HEDIS measures, flu immunization and osteoporosis management.

## Discussion

Medicare Advantage enrollees reported that price measures are their primary consideration when selecting a Medicare Advantage plan.^6^ Empirical research suggests that monthly premium is the most important price measure in enrollment decisions.^7^ Using data representative of the Medicare Advantage population, we investigated the extent to which enrollees experience higher quality care in higher premium plans.

We found that for the 78% of Medicare Advantage enrollees in plans with either a $0 or low monthly premium (≤$60), both patient experience and clinical quality were generally similar across these premium levels. Conversely, enrollees in moderately high (>$60 to <$120) and high (≥$120) premium plans reported better patient experiences and received higher-quality clinical care than those in $0 and low-premium plans. Although this finding was consistent across most individual measures and robust to model specification, the magnitude of these quality differences across premium categories were generally modest, particularly relative to the substantial quality differences in plans in the same premium category. Using the heuristic of 1, 3, and 5 points as small, medium, and large differences—with medium differences in quality being associated with higher plan disenrollment rates,^17^ the differences were almost all small-to-medium.

The exception to this otherwise consistent pattern of associations was an opposite pattern of worse clinical quality for osteoporosis management in higher premium plans. This negative relationship, which warrants additional study, was limited to for-profit plans.

Premiums are just 1 dimension of health care cost but are much easier to determine and understand than other dimensions that involve physician and hospital choice, patient disease burden, negotiated prices, and often complex plan cost-sharing features. These other costs are also usually not fixed but vary with individual health care use and require personalization to estimate total spending.^22^

The mechanism associating monthly premium and plan quality is unknown. In the employer-sponsored insurance market and on the individual exchanges, higher monthly premiums are associated with lower out-of-pocket costs at the point-of-care—lower deductibles and co-pays or co-insurance. In addition, low premium plans may have *narrow* networks (ie, smaller sets of in-network physicians) that may restrict patient choice and access. In Medicare Advantage, these relationships are altered by the Quality Bonus Program, whereby the Medicare Advantage plans with the highest past performance receive bonus payments to either provide additional enrollee benefits (eg, dental/vision coverage) or to lower the monthly premiums. If bonuses were to be used by high-quality plans to discount premiums, the associations between premium pricing and plan quality may weaken.^23^

Other aspects of enrollee cost-sharing, including deductibles, co-insurances, co-pays, out-of-pocket maximums, and subsidies may play a role in setting expectations of and producing quality. Network size may also play a role because smaller networks are likely to be associated with lower total plan costs and may have quality implications. One-third of Medicare Advantage enrollees are in plans with narrow physician networks,^24^ although only one-fifth of consumers feel positively about narrow networks when other options are presented.^3^ Conversely, 2 recent studies have found narrow networks to be associated with higher quality for Medicare Advantage plans.^25,26^ Medicare Advantage payment rates are unlikely to be a direct mechanism given that we controlled for HRR in all analyses, making all comparisons within regions where payment rates are the same.

### Limitations

These results may not hold for other measures of price, such as total out-of-pocket costs or for other dimensions of quality. Further research should examine these associations. Although we controlled for a number of plan characteristics, geographic area, and (for CAHPS measures) enrollee characteristics, unobserved factors related to quality may differ for enrollees of higher and lower premium plans. For instance, if those who enrolled in the highest premium plans have wealth and health advantages that are associated with higher quality, the results of our analyses would be an upper bound on the strength of the premium-quality association. For the CAHPS measures, survey nonresponse may lead to bias, although we note that response rates were very similar across premium levels (see eTable 9 in the Supplement). The sensitivity analyses are extensive which raises issues regarding multiple testing; to keep a strict type I error level for the entire family of tests in the appendices, a much lower *P* value may be considered.

## Conclusions

The findings of this retrospective study provide evidence of some positive association between price and quality for Medicare Advantage plans, particularly those with the highest premiums. However, given the highly variable quality within premium categories—high (and low) quality plans were found in each premium category—indicates that premium is at best a weak proxy for plan quality. Making plan quality information more accessible and salient to consumers remains key to reducing cost while improving quality.

## Notes

Supplement. eAppendix A. Methods: HEDIS–composite-like results through modeling eMethods. Figures 1 and 2 eTable 1. Random effect estimates for plan-level variance eAppendix B. Primary Model Full Results eTable 2A. Full model results for model summaries in Table 2 eTable 2B. Alternative model summaries to those shown in Table 2 eTable 3. Main model results for each component of the overall MA CAHPS composite eTable 4. Main model results for each HEDIS measure in the HEDIS composite model, Osteoporosis, and Flu Immunization eAppendix C. Sensitivity Analyses eTable 5. Main and supplementary model results for the MA CAHPS overall composite measure eTable 6. Main and supplementary model results for the HEDIS composite (excluding Osteoporosis Management and Influenza Immunization) eTable 7. Main and supplementary model results for the Osteoporosis Management HEDIS measure eTable 8. Main and supplementary model results for the Flu Immunization measure eTable 9. MA CAHPS response rates by premium level eFigure 1. Partial Residual Plot: Adjusted Relationship between CAHPS Composite and Continuous Monthly Premium eFigure 2. Partial Regression Plot: Adjusted Relationship between CAHPS Composite and Adjusted Monthly Premium eAppendix D. STROBE Reporting Recommendations
