# Supplementary material for: Association of Medicare Advantage Premiums With Measures of Quality and Patient Experience
Source: JAMA Health Forum. 2022 Aug 26;3(8):e222826. doi: 10.1001/jamahealthforum.2022.2826 (PMC9419011; doi:10.1001/jamahealthforum.2022.2826)
Supplement: Supplement. — eAppendix A. Methods: HEDIS–composite-like results through modeling eMethods. Figures 1 and 2 eTable 1. Random effect estimates for plan-level variance eAppendix B. Primary Model Full Results eTable 2A. Full model results for model summaries in Table 2 eTable 2B. Alternative model summaries to those shown in Table 2 eTable 3. Main model results for each component of the overall MA CAHPS composite eTable 4. Main model results for each HEDIS measure in the HEDIS composite model, Osteoporosis, and Flu Immunization eAppendix C. Sensitivity Analyses eTable 5. Main and supplementary model results for the MA CAHPS overall composite measure eTable 6. Main and supplementary model results for the HEDIS composite (excluding Osteoporosis Management and Influenza Immunization) eTable 7. Main and supplementary model results for the Osteoporosis Management HEDIS measure eTable 8. Main and supplementary model results for the Flu Immunization measure eTable 9. MA CAHPS response rates by premium level eFigure 1. Partial Residual Plot: Adjusted Relationship between CAHPS Composite and Continuous Monthly Premium eFigure 2. Partial Regression Plot: Adjusted Relationship between CAHPS Composite and Adjusted Monthly Premium eAppendix D. STROBE Reporting Recommendations [file jamahealthforum-e222826-s001.pdf]

Supplemental Online Content

Haviland AM, Ma S, Klein DJ, Orr N, Elliott MN. Association of Medicare Advantage premiums with measures of quality and patient experience. *JAMA Health Forum.* 2022;3(8):e222826. doi:10.1001/jamahealthforum.2022.2826

eAppendix A. Methods: HEDIS–composite-like results through modeling

eMethods. Figures 1 and 2

eTable 1. Random effect estimates for plan-level variance

eAppendix B. Primary Model Full Results

eTable 2A. Full model results for model summaries in Table 2

eTable 2B. Alternative model summaries to those shown in Table 2

eTable 3. Main model results for each component of the overall MA CAHPS composite

eTable 4. Main model results for each HEDIS measure in the HEDIS composite model, Osteoporosis, and Flu Immunization

eAppendix C. Sensitivity Analyses

eTable 5. Main and supplementary model results for the MA CAHPS overall composite measure

eTable 6. Main and supplementary model results for the HEDIS composite (excluding Osteoporosis Management and Influenza Immunization)

eTable 7. Main and supplementary model results for the Osteoporosis Management HEDIS measure

eTable 8. Main and supplementary model results for the Flu Immunization measure

eTable 9. MA CAHPS response rates by premium level

eFigure 1. Partial Residual Plot: Adjusted Relationship between CAHPS Composite and Continuous Monthly Premium

eFigure 2. Partial Regression Plot: Adjusted Relationship between CAHPS Composite and Adjusted Monthly Premium

eAppendix D. STROBE Reporting Recommendations

This supplemental material has been provided by the authors to give readers additional information about their work.

© 2022 Haviland et al. *JAMA Health Forum.*

Complete List of Supplemental Material

1. Appendix A: Methods Appendix
    a. HEDIS – composite-like results through modeling
    b. Methods for Figures 1 & 2
    c. eTable 1. Random effect estimates for plan-level variance:
2. Appendix B: Primary Model Full Results
    a. eTable 2a. Full model results for model summaries in Table 2
    b. eTale 2b. Alternative model summaries to those shown in Table 2
    c. eTable 3. Main model results for each component of the overall MA CAHPS composite
    d. eTable 4. Main model results for each HEDIS measure in the HEDIS composite model, Osteoporosis, and Flu Immunization
3. Appendix C: Sensitivity Analyses
    a. Part 1: Sensitivity to variable selection; results of secondary models
        i. eTable 5. Main and supplementary model results for the MA CAHPS overall composite measure
        ii. eTable 6. Main and supplementary model results for the HEDIS composite (excluding Osteoporosis Management and Influenza Immunization)
        iii. eTable 7. Main and supplementary model results for the Osteoporosis Management HEDIS measure
        iv. eTable 8. Main and supplementary model results for the Flu Immunization measure

    b. Part 2: Sensitivity regarding premium level
        i. eTable9. MA CAHPS response rates by premium level
        ii. eFigure 1. Partial Residual Plot: Adjusted Relationship between CAHPS Composite and Continuous Monthly Premium
        iii. eFigure2. Partial Regression Plot: Adjusted Relationship between CAHPS Composite and Adjusted Monthly Premium

© 2022 Haviland et al. *JAMA Health Forum.*

Appendix A: Methods Appendix:

Part 1: HEDIS – composite-like results through modeling

Because HEDIS measures apply to subsets of enrollees meeting specific eligibility criteria, each enrollee may only be eligible for one or a few measures, precluding creation of a composite measure at the enrollee level. To summarize the mean associations between premiums and quality across HEDIS measures included in this analysis, an analytic file was created with one record per person per eligible measure, such that each enrollee contributes zero to eight records, depending on the number of measures for which the enrollee was eligible. Some HEDIS measures have eligibility criteria which are much more often met than others; for example, eligibility for breast cancer screening is most common of the analyzed measures. So that all HEDIS measures count equally when summarizing the mean associations with premiums, plan-measure-level weights were created with values equal to the number of plan enrollees eligible for the most common measure (usually breast cancer screening) relative to the number eligible for the measure. We excluded the HEDIS measure on osteoporosis management in women who had a fracture from this composite as preliminary analyses showed a quite different relationship with premiums than other HEDIS measures. Results for this measure are shown separately and presented along with the summary results for the remaining eight HEDIS measures.
The HEDIS flu immunization receipt measure is collected through the Medicare CAHPS survey and so the data has a different structure. For this reason, flu immunization results are shown separately and not included in the pooled HEDIS summary.

© 2022 Haviland et al. *JAMA Health Forum.*

Part 2: Methods for Figures 1 & 2

CAHPS DATA Plan-level Standard Deviations within Premium Level

For the CAHPS composite, plan-level standard deviations were estimated separately for each premium category – allowing for them to differ. They were estimated using mixed linear regression within each premium category modeling the CAHPS composite with a random effect for plan and with the MCAHPS weight applied at the individual level. As with the primary models used to estimate premium effects, the model included fixed effects for hospital referral region, plan type, SNP type, and enrollee case-mix adjustors. Plan-level standard deviation was calculated as the square root of the random effect variance component estimate for the MA plan.

HEDIS DATA Plan-level Standard Deviations within Premium Level

For the combined HEDIS measures (those included in the HEDIS composite), as with CAHPS, plan-level standard deviation was estimated with mixed linear regression within each premium category modeling the outcome with a random effect for plan and with the measure weight applied at the plan level. As with the primary models used to estimate premium effects, the model included fixed effects for measure, plan type, and SNP type. Due to the large number of observations (over 3 million) and complexity of the model, these models would not converge with fixed effects for hospital referral region (which has many levels). To relieve some computational resources, in the model we replaced hospital referral region with the predicted probability of passing from a linear regression model where hospital referral region was the sole fixed effect; this model also employed the plan-level measure weight. With that change, the model converged for three of the four premium categories. For the >$0 – 60 category, in order to achieve convergence, we repeatedly fit the model using a random 20% sample of large plans (1,000 or more enrollees) and all smaller plans. We repeated that model with 20 total runs each using a different random sample. For the figure (and the table below) the estimate of plan-level variance was the mean of the estimates from these 20 models.Random effect estimates for both CAHPS and HEDIS models estimating plan-level variance are shown in the following table.

© 2022 Haviland et al. *JAMA Health Forum.*

eTable 1. Random effect estimates for plan-level variance:

Premium level	Plan-level variance estimate	Plan-level variance SE	Plan-level SD estimate (square root of variance)
**CAHPS**			
$0	79.53	22.98	8.92
>$0 – 60	55.18	9.18	7.43
>$60 – <$120	70.4	23.53	8.39
$120+	15.97	3.81	4.00
**HEDIS**			
$0	41.74	4.22	6.46
>$0 – 60	50.16	4.59	7.08
>$60 – <$120	30.48	4.17	5.52
$120+	23.39	4.64	4.84

© 2022 Haviland et al. *JAMA Health Forum.*

Appendix B: Primary Model Full Results

eTable 2a. Full model results for model summaries in Table 2

	CAHPS composite N = 166,988 R^2 = 0.057			Pooled HEDIS N = 3,192,511 R^2 = 0.063			Flu shot N = 161,411 R^2 = 0.038			Osteoporosis N = 77,054 R^2 = 0.054		
	b	se	p	b	se	p	b	se	p	b	se	p
Monthly premium (vs $0)												
>$0 - $60	2.18	0.34	<.0001	0.32	0.47	0.51	1.94	0.77	0.01	-3.60	1.54	0.02
>$60 - <$120	0.30	0.24	0.22	1.43	0.58	0.01	6.14	0.95	<.0001	-6.41	1.74	0.0002
$120+	1.44	0.34	<.0001	3.29	0.60	<.0001	10.32	1.00	<.0001	-6.14	1.81	0.0007
Plan Type (vs. HMO)												
HMO-POS	-0.64	0.54	0.24	---	---	---	0.26	1.13	0.82	---	---	---
Local PPO	-0.79	0.31	0.01	-1.08	0.49	0.03	-2.24	0.99	0.02	-5.17	1.35	0.0001
PFFS	0.15	0.81	0.86	-4.45	1.04	<.0001	-4.13	1.78	0.02	-11.62	1.91	<.0001
Regional PPO	-0.80	0.31	0.01	-3.45	0.82	<.0001	-3.83	1.47	0.009	-8.09	2.89	0.005
SNP Type (vs. non-SNP)												
Chronic	0.59	0.59	0.32	-0.77	0.76	0.31	3.76	1.74	0.03	11.77	2.96	<.0001
Dual eligible	0.64	0.48	0.18	-0.49	0.79	0.54	-2.12	1.00	0.03	2.40	2.65	0.36
Institutional	---	---	---	-2.32	1.25	0.07	---	---	---	-22.19	4.38	<.0001
Age (vs 70-74)												
18-64	-1.47	0.36	<.0001	---	---	---	---	---	---	---	---	---
65-69	-1.13	0.26	<.0001	---	---	---	---	---	---	---	---	---
75-79	-0.03	0.23	0.88	---	---	---	---	---	---	---	---	---
80-84	-0.50	0.33	0.13	---	---	---	---	---	---	---	---	---
85+	-0.29	0.32	0.37	---	---	---	---	---	---	---	---	---
Education (vs. HS grad)												
< 8th grade	-1.42	0.44	0.001	---	---	---	---	---	---	---	---	---
Some HS	-0.63	0.33	0.06	---	---	---	---	---	---	---	---	---
Some college	-0.51	0.22	0.02	---	---	---	---	---	---	---	---	---
College grad	-0.49	0.29	0.09	---	---	---	---	---	---	---	---	---
> College grad	-1.32	0.32	<.0001	---	---	---	---	---	---	---	---	---
General health (vs. good)												
Excellent	0.46	0.44	0.29	---	---	---	---	---	---	---	---	---
Very good	0.86	0.22	0.0001	---	---	---	---	---	---	---	---	---
Fair	-0.78	0.23	0.0009	---	---	---	---	---	---	---	---	---

© 2022 Haviland et al. *JAMA Health Forum.*

	CAHPS composite<br>N = 166,988<br>R^2 = 0.057			Pooled HEDIS<br>N = 3,192,511<br>R^2 = 0.063			Flu shot<br>N = 161,411<br>R^2 = 0.038			Osteoporosis<br>N = 77,054<br>R^2 = 0.054		
	**b**	**se**	**p**	**b**	**se**	**p**	**b**	**se**	**p**	**b**	**se**	**p**
Poor	-2.24	0.45	<.0001	---	---	---	---	---	---	---	---	---
Mental health (vs. good)												
Excellent	4.61	0.27	<.0001	---	---	---	---	---	---	---	---	---
Very good	1.71	0.24	<.0001	---	---	---	---	---	---	---	---	---
Fair	-1.25	0.31	<.0001	---	---	---	---	---	---	---	---	---
Poor	-3.45	0.56	<.0001	---	---	---	---	---	---	---	---	---
Any proxy assistance	-0.39	0.33	0.23	---	---	---	---	---	---	---	---	---
Answer proxy assistance	1.50	0.56	0.008	---	---	---	---	---	---	---	---	---
Medicaid eligibility	-0.86	0.39	0.03	---	---	---	---	---	---	---	---	---
Low Income Subsidy	-0.08	0.44	0.86	---	---	---	---	---	---	---	---	---
Measure (vs. Breast Cancer Screening)												
Drug therapy for rheumatoid arthritis	---	---	---	2.49	0.36	<.0001	---	---	---	---	---	---
Adult BMI Assessment	---	---	---	21.15	0.37	<.0001	---	---	---	---	---	---
Colorectal cancer screening	---	---	---	-0.56	0.33	0.09	---	---	---	---	---	---
Controlling High Blood Pressure	---	---	---	1.35	0.58	0.02	---	---	---	---	---	---
Diabetes blood sugar control	---	---	---	6.32	0.42	<.0001	---	---	---	---	---	---
Diabetes eye exam	---	---	---	-1.82	0.41	<.0001	---	---	---	---	---	---
Diabetes nephropathy	---	---	---	21.00	0.32	<.0001	---	---	---	---	---	---

All results are from linear regression models with outcomes ranging from 0-100 and with standard errors adjusted for clustering on plan.
All models additionally control for hospital referral region (HRR), results not shown.
MA CAHPS composite and flu shot models employ the individual-level MA CAHPS weight.
HEDIS pooled model employs a plan-level weight designed so that all HEDIS measures would count equally, given that respective eligibility for different measures varies by plan. The N of 3,192,511 represents outcomes; enrollees s may contribute more than one observation.
In CAHPS data, plan type has 5 categories, including separate categories for HMO and HMO-POS. In HEDIS, plan type has 4 levels, where HMO and HMO-POS are combined.
In CAHPS data, there are two types of Special Needs Plans (chronic and dual-eligible). In HEDIS data, institutional SNPs are present as a third type.

Abbreviations:
HMO = Health Maintenance Organization
HMO-POS = HMO with added Point of Sale benefits

© 2022 Haviland et al. *JAMA Health Forum.*

PPO = Preferred Provider Organization
PFFS = Private Fee for Service
SNP = Special Needs Plan
HS = High School
BMI = Body Mass Index

© 2022 Haviland et al. *JAMA Health Forum.*

eTable 2b. Alternative model summaries to those shown in Table 2

Adjusted mean (standard error)

Outcome	N	$0	>$0-60	>$60-<$120	$120+
MA CAHPS composite	166,988	82.27 (0.18)	82.57 (0.15)	83.71 (0.28)[cf]	84.46 (0.29)[cg]
HEDIS composite	3,192,511	81.34 (0.12)	81.66 (0.29)	82.77 (0.48)[ad]	84.63 (0.51)[ch]
HEDIS Osteoporosis	77,054	44.38 (1.08)	40.78 (0.92)[a]	37.97 (1.18)[c]	38.25 (1.35)[c]
Flu Immunization	161,411	68.55 (0.51)	70.49 (0.51)[a]	74.69 (0.77)[cf]	78.87 (0.84)[ci]

[a] p<0.05 [c] p<0.001 vs. $0 premium category
[d] p<0.05 [f] p<0.001 vs. >$0-$60 premium category
[g] <0.05 [h] p<0.001 [i] p<0.0001 vs. >$60-<$120 premium category
p<0.0001 for 3df block test of premium categories for MA CAHPS composite, HEDIS composite, and Flu shot measure model; p=0.001 for HEDIS Osteoporosis model.
All results are from linear regression models with standard errors adjusted for clustering on plan.
MA CAHPS composite and Flu shot models control for HR region, plan type, and SNP type, and employ the individual-level CAHPS weight. The CAHPS composite model additionally controls for case-mix adjustors.
HEDIS composite and Osteoporosis models control for HR region, plan type, and SNP type.
The HEDIS composite model employs a plan-level weight designed so that all HEDIS measures count equally, given that respective eligibility for different measures varies by plan.
The N of 3,192,511 represents person-outcomes; enrollees may contribute more than one observation

Sample sizes for the MA CAHPS survey are set by CMS to achieve sufficient reliability for reporting and pay-for-performance at the MA contract level.
Sample sizes for the HEDIS measures are set by CMS to achieve sufficient reliability and pay-for-performance at the MA contract level.

© 2022 Haviland et al. *JAMA Health Forum.*

eTable 3. Main model results for each component of the overall MA CAHPS composite

Coefficient (standard error)

Outcome	N	Monthly premium category				
		>$0-60 vs. $0	>$60-<$120 vs. $0	$120+ vs. $0	>$60-120 vs. >$0-60	$120+ vs. >$60-120
Getting needed drugs	154,223	-0.49 (0.32)	0.47 (0.42)	1.79 (0.40)[c]	0.96 (0.42)[a]	1.32 (0.46)[b]
Get needed care	163,239	0.52 (0.34)	1.67 (0.44)[c]	2.52 (0.47)[c]	1.15 (0.41)[b]	0.85 (0.50)
Get care quickly	151,046	0.70 (0.39)	2.04 (0.51)[c]	2.78 (0.53)[c]	1.34 (0.49)[b]	0.73 (0.56)
Customer service	87,916	-0.05 (0.44)	1.10 (0.84)	0.16 (0.62)	1.15 (0.74)	-0.94 (0.81)
Care coordination	142,245	0.44 (0.32)	1.11 (0.37)[b]	2.04 (0.37)[c]	0.67 (0.38)	0.93 (0.41)[a]

[a] $p<0.05$ [b] $p<0.01$ [c] $p<0.001$
$p<0.0001$ for 3df block test of monthly premium categories for all outcomes except for customer service ($p=0.49$).
Models control for case-mix adjustors, health referral region, plan type, and special needs plan type. Models employ the MA CAHPS weight and standard errors are adjusted for clustering on plan.
PDP = prescription drug plan

© 2022 Haviland et al. *JAMA Health Forum.*

eTable 4. Main model results for each HEDIS measure in the HEDIS composite model, Osteoporosis, and Flu Immunization

			Coefficient (standard error) vs $0					
			Monthly premium category					3df premiums p-value
Outcome	N	Overall pass rate	>$0-60	>$60-<$120	$120+	>$60-<$120 vs. >$0-60	$120+ vs. >$60-<$120	
Adult BMI assessment	156,488	93.7%	-0.79 (.0.68)	0.92 (0.91)	0.89 (0.89)	1.71 (0.90)	-0.03 (0.94)	.12
Breast Cancer screening	2,139,422	75.5%	-0.15 (0.49)	1.26 (0.57)[a]	3.83 (0.67)[c]	1.41 (0.49)[b]	2.57 (0.61)[c]	<.0001
Colorectal Cancer screening	217,372	74.7%	2.89 (1.03)[b]	6.55 (1.25)[c]	9.97 (1.41)[c]	3.67 (1.09)[c]	3.42 (1.15)[b]	<.0001
Controlling Blood pressure	126,014	66.1%	-1.61 (1.49)	-1.35 (2.18)	2.39 (1.81)	0.26 (2.44)	3.74 (2.06)	.06
Diab / HbA1c control	155,432	77.2%	-0.96 (1.21)	1.78 (1.39)	1.37 (1.53)	2.74 (1.42)	-0.41 (1.62)	.23
Diab / nephropathy	199,464	96.2%	0.04 (0.17)	0.43 (0.25)	0.66 (0.29)	0.39 (0.24)	0.22 (0.32)	.07
Diab / eye exam	156,646	72.6%	1.54 (0.96)	3.91 (1.18)[b]	7.16 (1.33)[c]	2.37 (1.19)[a]	3.24 (1.32)[a]	<.0001
Drug therapy for arthritis	160,711	77.4%	1.17 (0.57)[a]	1.73 (0.56)[b]	2.33 (0.73)[b]	0.56 (0.53)	0.60 (0.68)	.004
Osteoporosis Management	77,054	41.4%	-3.60 (1.54)[a]	-6.42 (1.74)[c]	-6.14 (1.81)[c]	-2.81 (1.45)	0.27 (1.67)	.001
Flu immunization	161,411	70.9%	1.94 (0.77)[a]	6.14 (0.95)[c]	10.32 (1.00)[c]	4.20 (0.91)[c]	4.19 (1.03)[c]	<.0001

[a] p<0.05 [b] p<0.01 [c] p<0.001
Models control for health referral region, plan type, and special needs plan type.
Standard errors are adjusted for clustering at the plan level.
Measures above *osteoporosis management* were included in the pooled model.
Flu immunization model employs individual-level CAHPS sampling weights.

© 2022 Haviland et al. *JAMA Health Forum.*

Appendix C: Sensitivity Analyses

Part 1: Sensitivity to variable selection; results of secondary models

As shown in eTables 5-8, for all these measures, the relationship between plan premium category and quality is similar although slightly weaker in the baseline model (Model 0) that does not control for plan structure relative to the main models that include controls for plan structure. For all these measures, the main model findings are robust to the inclusion of an additional measure of plan generosity – the maximum allowed out-of-pocket amount – in Model 2, where no statistical significance levels change, and magnitudes are similar to Model 1. Model 3 tests for differences in the association between premium and quality in for-profit MA plans versus not-for-profit MA plans. We do not detect different associations by profit status between premium and overall composite patient experience, the pooled HEDIS clinical quality measure, or receipt of a *flu immunization*. In contrast, for the *osteoporosis management* measure the negative relationship between receipt of recommended care and premium level holds only for plans that are for-profit ($p < 0.001$). For not-for-profit plans, *osteoporosis management* levels are similar for all but the highest premium level plans where levels are higher ($p < 0.01$) than for other plans. In Model 4 we replace HRR with county and find results are similar to those in Model 1 both with regard to point estimates and statistical significance levels. In Model 5 we omit MA enrollees who were also enrolled in Medicaid and again find similar results.

© 2022 Haviland et al. *JAMA Health Forum.*

eTable 5. Main and supplementary model results for the MA CAHPS overall composite measure (N=166,988)

Monthly premium category	Model 0 (HRR+ CMAs)			Model 1 (add plan type)			Model 2 (add MOOP)			Model 3 (model 1 + premium X for profit)		
	**beta**	**se**	**p**	**beta**	**se**	**p**	**beta**	**se**	**p**	**beta**	**se**	**p**
$0 (reference)	---	---	---	---	---	---	---	---	---	---	---	---
>$0-60	0.36	0.24	0.14	0.30	0.24	0.22	0.35	0.24	0.14	0.28	0.27	0.31
>$60 - <$120	1.22	0.34	0.0004	1.44	0.34	<.0001	1.42	0.34	<.0001	1.28	0.44	0.004
$120+	1.96	0.33	<.0001	2.18	0.34	<.0001	2.24	0.35	<.0001	1.91	0.47	<.0001
>$60-<$120 vs. >$0-60	0.86	0.31	0.006	1.14	0.31	0.0002	1.07	0.32	0.0009	1.00	0.35	0.004
$120+ vs. >$60-<$120	0.73	0.36	0.04	0.75	0.37	0.04	0.82	0.38	0.03	0.63	0.37	0.09
For-profit X premium ($)										0.00[#]	0.00[#]	0.85
MOOP (ref=<$3500)**												
$3,500-6000							0.48	0.36	0.18			
>$6,000							-0.57	0.38	0.13			
Missing							-7.57	5.38	0.16			

Monthly premium category	Model 4 (replace HRR with county)			Model 5 (remove those with dual status, N=121,137)		
	**beta**	**se**	**p**	**beta**	**se**	**p**
$0 (reference)	---	---	---	---	---	---
>$0-60	0.41	0.26	0.11	0.49	0.25	0.053
>$60 - <$120	1.58	0.36	<.0001	1.50	0.36	<.0001
$120+	2.15	0.34	<.0001	1.97	0.32	<.0001
>$60-<$120 vs. >$0-60	1.16	0.34	0.0007	1.00	0.31	0.001
$120+ vs. >$60-<$120	0.58	0.36	0.11	0.47	0.35	0.17

p<0.001 for 3df block test of monthly premium categories for all models.
** MOOP = Maximum Out Of Pocket expenses. Though these 3 comparisons vs. <$3500 are n/s, 3df block test is significant, p=0.002.
Models employ the MA CAHPS survey and non-response weights, and standard errors are adjusted for clustering on plan.
# Exact coefficient (standard error) = 0.00083 (0.0045).
HRR = health referral region. CMAs = case-mix adjusters.

© 2022 Haviland et al. *JAMA Health Forum.*

eTable 6. Main and supplementary model results for the HEDIS composite (excluding Osteoporosis Management and Influenza Immunization)

Monthly premium category	Model 0 (HRR)			Model 1 (add plan type)			Model 2 (add MOOP)			Model 3 (model 1 + premium X for profit)		
	beta	se	p	beta	se	p	beta	se	p	beta	se	p
$0 (reference)	---	---	---	---	---	---	---	---	---	---	---	---
>$0-60	-0.02	0.51	0.97	0.32	0.47	0.51	-0.05	0.43	0.91	-0.02	0.56	0.98
>$60 - <$120	0.41	0.57	0.47	1.43	0.58	0.01	1.16	0.58	0.04	0.58	0.98	0.55
$120+	2.79	0.63	<.0001	3.29	0.60	<.0001	2.83	0.62	<.0001	1.31	1.70	0.44
>$60-<$120 vs. >$0-60	0.43	0.53	0.41	1.11	0.55	0.04	1.21	0.55	0.03	0.60	0.73	0.41
$120+ vs. >$60-<$120	2.39	0.61	<.0001	1.86	0.58	0.001	1.67	0.57	0.003	0.73	0.99	0.46
For-profit X premium ($)										0.01	0.01	0.47
MOOP (ref=<$3500)**												
$3,500-6000							-1.05	0.56	0.06			
>$6,000							-1.01	0.61	0.09			
Missing							3.70	0.92	<.0001			

Monthly premium category	Model 4 (replace HRR with county)			Model 5 (remove those with dual status)		
	beta	se	p	beta	se	p
$0 (reference)	---	---	---	---	---	---
>$0-60	0.23	0.53	0.67	0.22	0.48	0.64
>$60 - <$120	1.28	0.64	0.04	1.25	0.55	0.02
$120+	3.43	0.59	<.0001	2.71	0.58	<.0001
>$60-<$120 vs. >$0-60	1.06	0.63	0.09	1.03	0.54	0.06
$120+ vs. >$60-<$120	2.14	0.62	0.0006	1.46	0.55	0.008

p<0.0001 for 3df block test of monthly premium categories for all models except model 4, p=0.77
Models employ plan-measure-level weights so that all eight HEDIS outcomes have equal influence within plans.
Standard errors are adjusted to account for clustering on plan.
** p<0.0001 for 3df block test of Maximum Out Of Pocket expenses.
HRR = health referral region.

© 2022 Haviland et al. *JAMA Health Forum.*

eTable 7. Main and supplementary model results for the Osteoporosis Management HEDIS measure (N=77,054)

Monthly premium category	Model 0 (HRR)			Model 1 (add plan type)			Model 2 (add MOOP)			Model 3 (model 1 + premium X for profit)		
	beta	se	p	beta	se	p	beta	se	p	beta	se	p
$0 (reference)	---	---	---	---	---	---	---	---	---	---	---	---
>$0-60	-3.60	1.54	0.02	-3.99	1.49	0.007	-4.26	1.46	0.004	0.35	1.72	0.84
>$60 - <$120	-6.41	1.74	0.0002	-9.30	1.54	<.0001	-7.01	1.68	<.0001	2.75	2.67	0.30
$120+	-6.14	1.81	0.0007	-7.85	1.72	<.0001	-7.05	1.81	0.0001	10.67	4.34	0.01
>$60-<$120 vs. >$0-60	-2.81	1.45	0.053	-5.32	1.45	0.0002	-2.76	1.39	0.047	2.40	1.89	0.20
$120+ vs. >$60-<$120	0.27	1.67	0.87	1.45	1.75	0.41	-0.03	1.61	0.98	7.92	2.58	0.002
For-profit X premium ($)										-0.13	0.03	<.0001
MOOP (ref=<$3500)**												
$3,500-6000							-2.42	1.61	0.13			
>$6,000							-3.88	1.70	0.02			
Missing							11.91	3.88	0.002			

Monthly premium category	Model 4 (replace HRR with county)			Model 5 (remove those with dual status, N=64,183)		
	beta	se	p	beta	se	p
$0 (reference)	---	---	---	---	---	---
>$0-60	-4.00	1.58	0.01	-3.76	1.57	0.02
>$60 - <$120	-6.03	1.74	0.0005	-6.26	1.72	0.0003
$120+	-5.76	1.79	0.001	-7.12	1.81	<.0001
>$60-<$120 vs. >$0-60	-2.04	1.51	0.18	-2.50	1.42	0.08
$120+ vs. >$60-<$120	0.27	1.70	0.87	-0.86	1.62	0.60

p<0.001 for 3df block test of monthly premium categories for all models except for model 4, p=0.01
Standard errors are adjusted to account for clustering on plan.
** p=0.0009 for 3df block test of Maximum Out Of Pocket expenses.
HRR = health referral region.

© 2022 Haviland et al. *JAMA Health Forum.*

eTable 8. Main and supplementary model results for the Flu Immunization measure (N=161,411)

Monthly premium category	Model 0 (HRR+ CMAs)			Model 1 (add plan type)			Model 2 (add MOOP)			Model 3 (model 1 + premium X for profit)		
	beta	se	p	beta	se	p	beta	se	p	beta	se	p
$0 (reference)	---	---	---	---	---	---	---	---	---	---	---	---
>$0-60	0.95	0.74	0.20	1.94	0.77	0.01	2.04	0.77	0.008	1.24	0.79	0.12
>$60 - <$120	4.93	0.95	<.0001	6.14	0.95	<.0001	6.17	0.95	<.0001	4.53	1.08	<.0001
$120+	9.62	1.01	<.0001	10.32	1.00	<.0001	10.78	1.04	<.0001	8.33	1.13	<.0001
>$60-<$120 vs. >$0-60	3.98	0.88	<.0001	4.20	0.91	<.0001				3.29	0.96	0.0006
$120+ vs. >$60-<$120	4.69	1.06	<.0001	4.19	1.03	<.0001				3.80	1.03	0.0002
For-profit X premium ($)										0.03	0.01	0.01
MOOP (ref=<$3500)**												
$3,500-6000							2.70	0.86	0.002			
>$6,000							0.05	1.13	0.97			
Missing							-4.31	10.78	0.69			

Monthly premium category	Model 4 (replace HRR with county)			Model 5 (remove those with dual status, N=118,060)		
	beta	se	p	beta	se	p
$0 (reference)	---	---	---	---	---	---
>$0-60	1.82	0.76	0.02	2.82	0.88	0.001
>$60 - <$120	6.16	0.96	<.0001	6.36	1.00	<.0001
$120+	10.07	0.98	<.0001	10.59	1.08	<.0001
>$60-<$120 vs. >$0-60	4.34	0.91	<.0001	3.54	1.01	0.0005
$120+ vs. >$60-<$120	3.91	1.04	0.0002	4.23	1.07	<.0001

p<0.0001 for 3df block test of monthly premium categories for all models.
** p=0.001 for 3df block test of Maximum Out Of Pocket expenses.
Models employ the MA CAHPS survey and non-response weights, and standard errors are adjusted for clustering on plan.
HRR = health referral region. CMAs = case-mix adjusters.

© 2022 Haviland et al. *JAMA Health Forum.*

Part 2: Sensitivity analyses regarding premium level

eTable 9. MA CAPHS response rates by premium level.

Monthly premium category	Response rate
$0	42.9%
>$0-60	42.7%
>$60 - <$120	47.2%
$120+	47.0%

Item Response/Missing Values in MA CAHPS analytic data set:
In the analysis file of 168,750 survey respondents as defined in the Data section, there is no missing data for the enrollee level case-mix adjustors, nor for any of the other predictors in the models (e.g., contract-level characteristics), except for HRR. There were 3,269 (1.9%) records missing HRR, 99% of which (n=3,228) were in Puerto Rico. We created a new HRR code for PR, and another for the remaining cases where HRR was missing and the enrollee was not in Puerto Rico (n=41), and therefore all respondents in the analysis file had complete data and were included in the models. There was some missingness for MOOP (Maximum Out-of-Pocket cost) and we included that as a "missing" category in analysis & tables. The CAHPS composite has some missingness – it is missing for n=1,762 (1.0%), survey respondents who did not respond to any of the items in the composite, so they are excluded, and the models have N=166,988 (as stated in the tables). Missingness is higher for the individual items, and the composite is the mean of non-missing items.
The Flu Immunization measure has missingness; it is missing for n=7,339 (4.3%) of survey respondents. These survey respondents are omitted from the Flu Immunization models resulting in an N=161,411 (as stated in the tables).

Specification of the cut-points for Premium Levels
To check whether the results we show are likely to be sensitive to the cut points we used in defining the premium levels, we constructed both a partial residual plot (eFigure 1 below) and a partial regression plot (eFigure 2 below) illustrating the relationship between the CAHPS composite and the continuous premium measure, accounting for other model covariates.

To generate the data for the partial residual plot shown in eFigure 1, we ran a linear regression modeling the CAHPS composite employing individual-level sampling weights and adjusting standard errors for clustering on plan. Fixed effects were the plan characteristic, and case-mix adjustors described in the Methods section for the CAHPS primary model, plus HRR region, but without premiums. Residuals were retained from this model and then standardized to mean of zero and standard deviation of one. We then created a scatterplot with these standardized residuals on the y-axis and the continuous specification of the monthly premium on the x-axis. Due to the large number of data points, a loess curve was fitted to facilitate visualizing the relationship.

To generate the data for the partial regression plot shown in eFigure 2 we used the same data for the vertical axis as for the partial residual plot. For the horizontal axis data, we ran a linear regression with the continuous version of monthly premium as the outcome but otherwise the same as the model used to construct the vertical axis data. Residuals again were retained and standardized. We created the partial regression plot by plotted these two sets of residuals against each other.

The partial residual plot shows that the mean residual remains close to zero at all premium levels and there is no clear pattern of residuals increasing or decreasing as premiums change. There also is no evidence of jumps in the patient experience composite residuals at different thresholds of the premium. We selected the cut points for the premium levels used in these analyses based on ease of interpretation. The cut points for the levels are $0, $60, and $120. $60 per month is about $2 per day and $120 per month is about $4 per day. The partial regression plot does not show evidence of concerning outliers or high leverage points.

© 2022 Haviland et al. *JAMA Health Forum.*

eFigure 1. Partial Residual Plot: Adjusted Relationship between CAHPS Composite and Continuous Monthly Premium

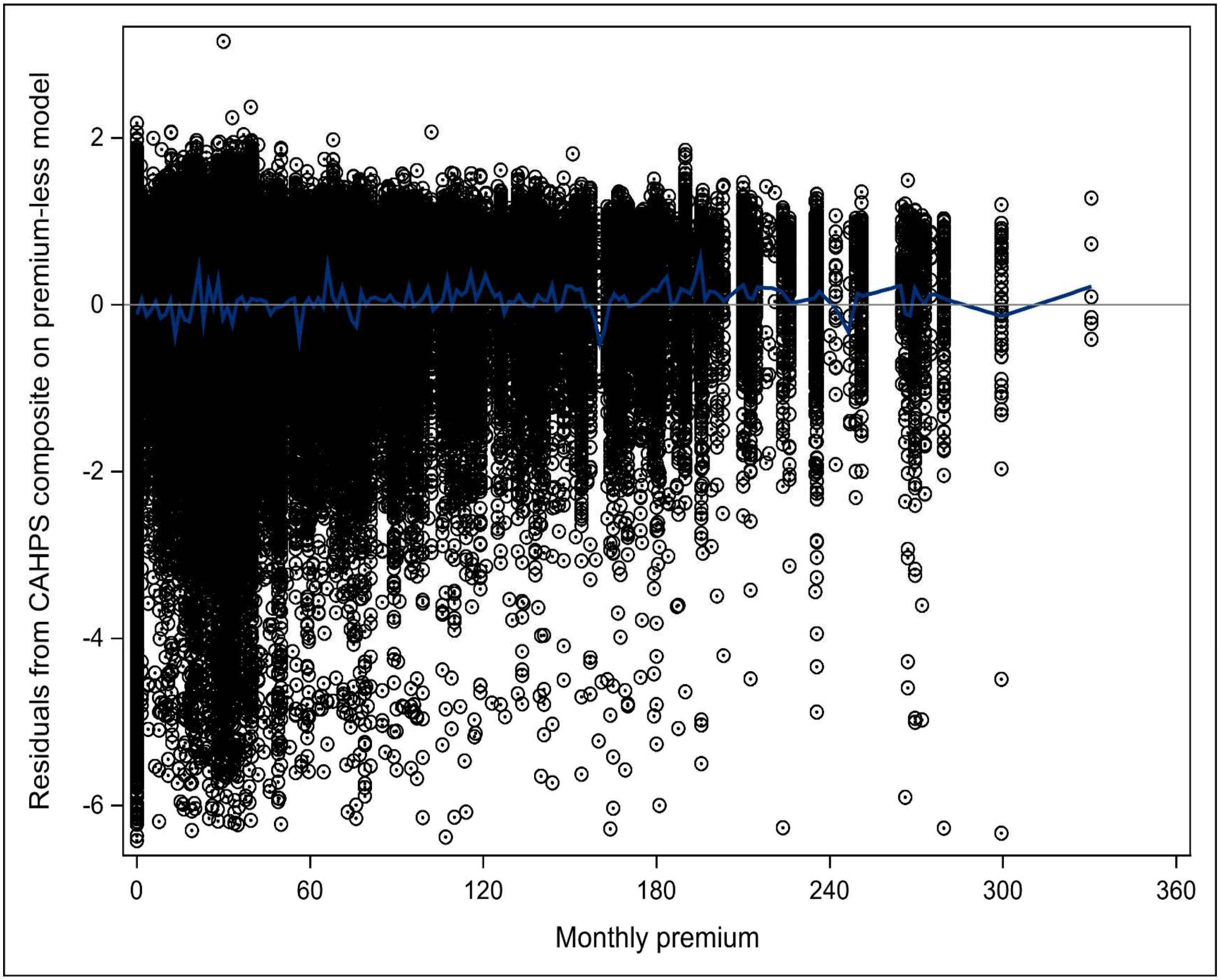

© 2022 Haviland et al. *JAMA Health Forum.*

eFigure 2. Partial Regression Plot: Adjusted Relationship between CAHPS Composite and Adjusted Monthly Premium

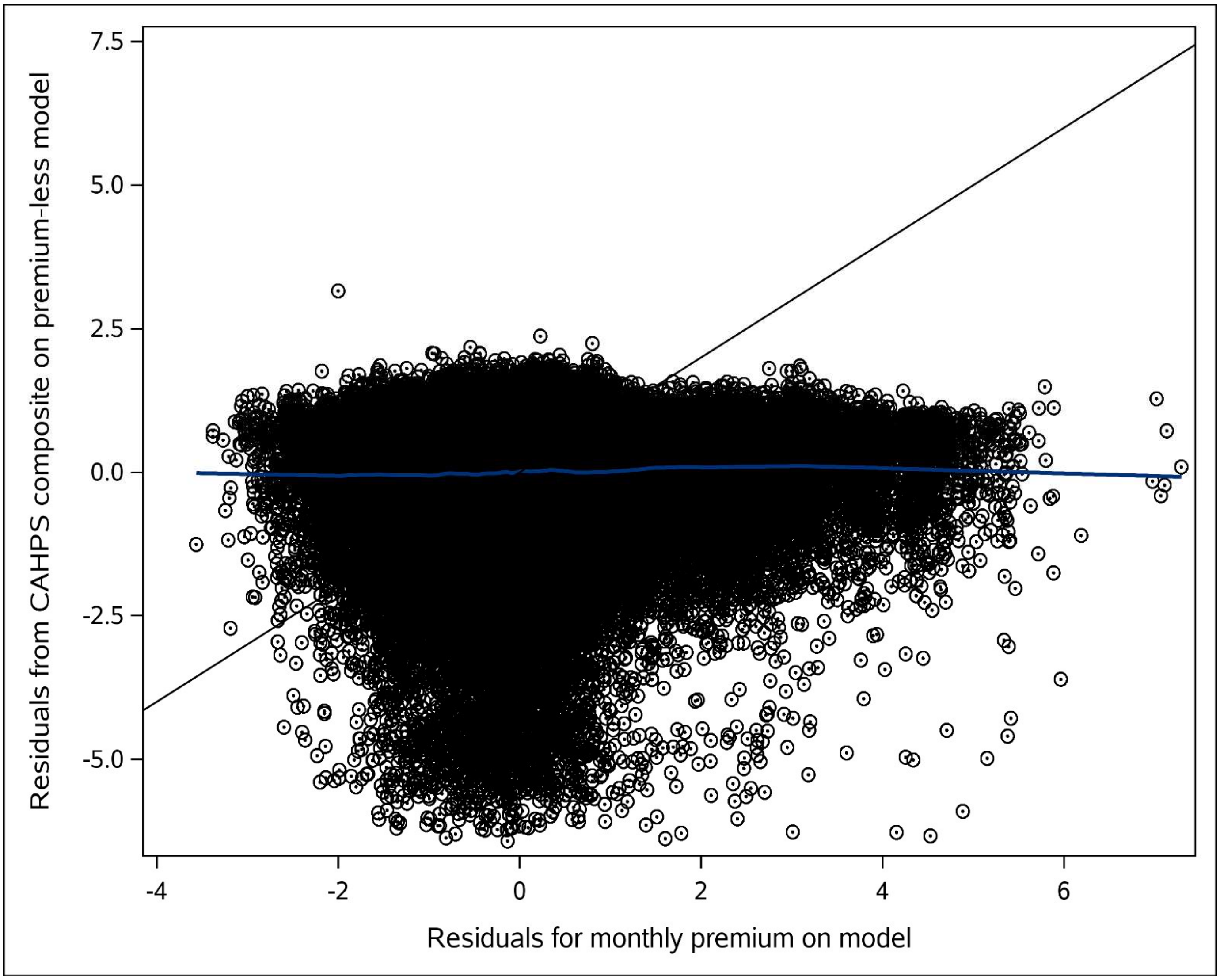

© 2022 Haviland et al. *JAMA Health Forum.*

Appendix D: STROBE Reporting Recommendations

Item	Reporting Recommendation	Location in Manuscript
1	Title and abstract: (a) Indicate the study's design with a commonly used term in the title or the abstract. (b) Provide in the abstract an informative and balanced summary of what was done and what was found	Abstract and Key Points
2	Introduction: Explain the scientific background and rationale for the investigation being reported	Introduction
3	Introduction: State specific objectives, including any prespecified hypotheses	Key Points: Question & Introduction final paragraph
4	Methods: Present key elements of study design early in the paper	Abstract: Design & Methods paragraph 4
5	Methods: Describe the setting, locations, and relevant dates, including periods of recruitment, exposure, follow-up, and data collection.	Methods paragraph 1
6	Methods: Give the eligibility criteria, and the sources and methods of selection of participants	Methods paragraph 2 & 6
7	Methods: Clearly define all outcomes, exposures, predictors, potential confounders, and effect modifiers. Give diagnostic criteria, if applicable.	Methods: Measures & Analysis sections
8	Methods: For each variable of interest, give sources of data and details of methods of assessment (measurement). Describe comparability of assessment methods if there is more than one group.	Methods paragraph 1 & Appendix C
9	Methods: Describe any efforts to address potential sources of bias.	Methods Analysis section
10	Methods: Explain how the study size was arrived at	eTable 2b
11	Methods: Explain how quantitative variables were handled in the analyses. If applicable, describe which groupings were chosen and why.	Methods Analysis section & Appendix A & Appendix C
12	Methods: (a) describe all statistical methods, including those used to control for confounding (b) describe any methods used to examine subgroups and interactions (c) explain how missing data were addressed (d) If applicable, describe analytical methods taking account of sampling strategy, (e) describe any sensitivity analyses	(a) Methods Analysis section & Appendix A<br>(b) Methods paragraph 10<br>(c) Appendix C<br>(d) Methods paragraph 2<br>(e) Methods paragraph 10 and Appendix C
13	Results: (a) Report numbers of individuals at each stage of study- e.g., numbers potentially eligible,	Methods paragraphs 2 & 3 and Appendix C

© 2022 Haviland et al. *JAMA Health Forum.*

Item	Reporting Recommendation	Location in Manuscript
	examined for eligibility, confirmed eligible, included in the study, completing follow-up, and analyzed (b) give reasons for non-participation at each stage (c) consider use of a flow diagram	
14	Results: (a) Give characteristics of study participants (e.g., demographic, clinical, social) and information on exposures and potential confounders (b) indicate number of participants with missing data for each variable of interest.	(a) Table 1 and Results paragraph 1<br>(b) Appendix C
15	Results: Report numbers of outcome events or summary measures	Figures 1 & 2, eTable 2b, eTable 3, eTable 4
16	Results: (a) give unadjusted estimates and, if applicable, confounder-adjusted estimates and their precision (e.g., 95% confidence interval). Make clear what confounders were adjusted for and why they were included (b) report category boundaries when continuous variables were categorized (c) if relevant, consider translating estimates of relative risk into absolute risk for a meaningful time period	(a) Table 2, eTable2b, Results paragraphs 2, 3, 4, 5. We do not present unadjusted estimates as any inferences regarding them are considered inappropriate (see National Cancer Institute, Division of Cancer Control & Population Sciences. (2020). "Case-mix Adjustment Guidance." https://healthcaredelivery.cancer.gov/seer-cahps/researchers/adjustment_guidance.html<br>(b) Premium category boundaries are stated each time in the tables and results. Sensitivity to these boundaries is investigated in Appendix C<br>(c) NA
17	Results: Report other analyses done-e.g., analyses of subgroups and interactions, and sensitivity analyses	All other analyses are shown in eTables 4 - 8
18	Discussion: Summarize key results with reference to study objectives	Discussion paragraphs 1 (key objective), 2, and 3 (summary of key results)
19	Discussion: Discuss limitations of the study, taking into account sources of potential bias or imprecision. Discuss both direction and magnitude of any potential bias.	Discussion: Limitations section. We discuss the direction of likely potential bias but do not have any way to estimate its magnitude. We do discuss the implications of bias in this direction for the interpretation of results.
20	Discussion: Give a cautious overall interpretation of results considering objectives, limitations, multiplicity of analyses, results from similar studies, and other relevant evidence	Discussion section overall and the Conclusions paragraph at the end in particular.
21	Discussion: Discuss the generalizability (external validity) of the study results	Discussion paragraph 1
22	Other information: Give the source of funding and the role of the funders for the present study and, if applicable, for the original study on which the present article is based.	Acknowledgements Section

© 2022 Haviland et al. *JAMA Health Forum.*
